# Refractory Immunological Thrombocytopenia Purpura and Splenectomy in Pregnancy

**DOI:** 10.1155/2015/216362

**Published:** 2015-12-22

**Authors:** Santiago Bernal-Macías, Laura-Marcela Fino-Velásquez, Felipe E. Vargas-Barato, Lucio Guerra-Galue, Benjamín Reyes-Beltrán, Adriana Rojas-Villarraga

**Affiliations:** ^1^Center for Autoimmune Diseases Research (CREA), School of Medicine and Health Sciences, Universidad del Rosario, Bogotá, Colombia; ^2^Surgery Department, School of Medicine and Health Sciences, Universidad del Rosario, Bogotá, Colombia; ^3^Surgery Department, Hospital Universitario Mayor-Mederi (HUM), Bogotá, Colombia; ^4^Gynaecology Department, Hospital Universitario Mayor-Mederi (HUM), Bogotá, Colombia

## Abstract

Thrombocytopenia is defined as a platelet count of less than 100,000 platelets per microlitre (mcL). Thrombocytopenia develops in approximately 6-7% of women during pregnancy and at least 3% of these cases are caused by immunological platelet destruction. Herein, we present a pregnant woman who develops at the first trimester autoimmune thrombocytopenia purpura associated with positive antiphospholipid antibodies. The disease was refractory to pharmacological treatments but had a favourable response to splenectomy. The patient carried the pregnancy to term without complication and gave birth to a healthy baby girl.

## 1. Introduction

The immunological thrombocytopenic purpura (ITP) is the accelerated destruction or inadequate platelet production mediated by autoantibodies; it is important to consider there are other causes of thrombocytopenia in pregnancy. Other causes include the following: preeclampsia, HELLP syndrome, thrombocytopenic thrombotic purpura, haemolytic uremic syndrome, congenital conditions, drugs (i.e., heparin and quinidine), infections (i.e., human immunodeficiency virus, hepatitis C, cytomegalovirus, and Epstein-Barr virus), lymphoproliferative disorders, bone marrow diseases, and autoimmune diseases (AD) (i.e., systemic lupus erythematosus (SLE) and antiphospholipid syndrome (APS)) [1–5].

In previously healthy women, ITP usually presents as an incidental finding in an asymptomatic woman. There are also a small percentage of cases that present with mucocutaneous bleeding manifestations [4].

## 2. Case

**Figure 1:**
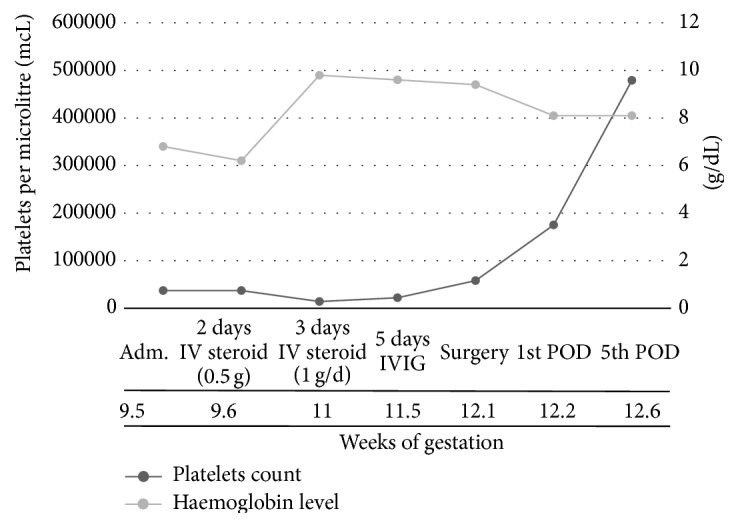
Evolution of platelets count and haemoglobin levels during pregnancy on different treatments. Adm.: admission; IV: intravenous; IVIG: intravenous immunoglobulins; POD: postoperative day.

A 24-year-old woman during week 9.5 of an uncomplicated pregnancy presented to the emergency department with sudden onset of a major epistaxis episode. Her platelet count was 15000 mcL (Figure 1). A multidisciplinary treatment approach was required and the patient was referred to a third-level hospital in December 2013.

The physical examination revealed an active epistaxis that was controlled by anterior nasal packing. The remaining examination was normal. The haemoglobin level was 6.2 g per deciliter (g/dL) and the platelet count was 37,000 platelets per microlitre (mcL). The direct antiglobulin test (Coombs test) was positive; however Evans' syndrome was ruled out based on the laboratory tests which came out negative to detect the presence of haemolysis (i.e., peripheral blood smear, reticulocytes production index, levels of serum lactate dehydrogenase, serum haptoglobin, and indirect bilirubin) and the standard autoimmune profile was negative except for positive IgG anticardiolipin antibodies (aCL) (titer of 47.8 GLP) and moderately positive lupus anticoagulant (LA) (LA1/LA2 ratio: 1.74).

The patient was diagnosed with ITP because she was previously healthy and had no prior history of any thrombosis event, foetal loss, preterm labour, or familiar autoimmunity suggesting APS, SLE, or AD. Consequently, the patient was treated with a transfusion of three units of packed red blood cells and corticosteroids orally and intravenously. The initial treatment included a one-day methylprednisolone bolus of 500 mg and then sustained doses of prednisolone of 50 mg/day.

After six days of treatment, the platelet count dropped to 2,000/mcL. Therefore, it was necessary to add a new course of methylprednisolone bolus of 1 g/day for three days. The patient received a transfusion of 18 units of platelets. The persistence of platelets under 30,000/mcL indicated a failure of corticosteroid treatment. Therefore, a five-day course of intravenous immunoglobulins (IVIG) of 0.4 g/kg/day was used.

Three weeks after disease onset, the patient experienced three additional episodes of epistaxis (all with less than 30,000/mcL platelets; one episode before the first day and two episodes day 0 and day 1 after course of IVIG) that required local control by otolaryngology. Due to the failure of pharmacological treatment, a multidisciplinary consensus between Gynaecology, Internal Medicine, General Surgery, and Rheumatology approved a laparoscopic splenectomy. The surgery was performed the next day and the patient had a platelet count of 18,000/mcL and required transfusion of 12 units of platelets before and during the surgery according with anaesthesiologist. The platelet count in the early postoperative period was 58,000/mcL.

The patient was closely monitored in the intensive care unit (ICU) for 4 days subsequent to the surgery. A rapid increase of platelet count is notorious the following postoperative day (POD), and the patient's count increased to 175,000/mcL on POD 1 and 479,000/mcL by POD 5.

The patient was discharged after 25 days and was in the 13th week of gestation. The pharmacological treatment with prednisolone 40 mg/day was gradually decreased. Additionally, the patient was treated with low molecular weight heparin during the last 8 weeks of gestation as directed by her gynaecologist. As a preventive strategy, the patient was vaccinated for encapsulated bacteria. The patient carried the pregnancy to term without complication and gave birth to a healthy baby girl.

## 3. Discussion

We here presented an unusual case of ITP associated with positive antiphospholipid antibodies during the first trimester of pregnancy. The patient required splenectomy due to the persistence of low platelet counts and haemorrhagic manifestations despite pharmacological treatment.

The pathogenetic role and the clinical importance of the presence of antiphospholipid antibodies (aPL) in patients with ITP are not clear. Diz-Küçükkaya et al. reported that 37.8% of cases had aPL in a cohort of patients with ITP followed up for 5 years. The authors did not identify any differences in platelet count or response to methylprednisolone [6]. Yang et al. found a similar prevalence of aPL (28.5%) in a cohort of patients with ITP [7] and Stasi et al. demonstrated higher prevalence of aPL (46.3%) in their cohort [8]. However, the series of patients with APS were evaluated for thrombocytopenia risk in the presence of aCL and the results showed that high titre of aCL IgG has a predictive value of 77% for thrombocytopenia [9].

The presence of LA is an important marker of thrombosis in patients with ITP. Thus, Diz-Küçükkaya et al. concluded that the persistent presence of aPL in patients with ITP is an important risk factor for the development of APS [6].

The patient did not fulfill the clinical criteria for APS (thrombotic events, recurrent foetal loss, and preterm birth before 34 weeks of gestation [10]). However, the clinical suspicion of APS alone or with concurrent AD (i.e., polyautoimmunity) is treated according to the APS International Consensus proposed in 2006 [11, 12]. Nevertheless, the presence of aPL and thrombocytopenia without prior thrombosis or foetal loss is not classified as APS [9].

The 2011 American Society of Haematology (ASH) determined there are no studies comparing different treatments or comparing treatment to nontreatment in pregnant women and all data are based on observational studies [5]. Corticosteroids (prednisolone and methylprednisolone: FDA category C) are the most effective and secure pharmacological group to decrease the activity of the disease in pregnant patients. However, side effects can be generated as hypertension, delayed foetal growth, and cleft lip or cleft palate [4, 5, 13, 14].

IVIG is category C according to FDA classification and is administered at 0.4 mg/kg/day for 5 days or 1 mg/kg/day for 2 days and achieves an acceptable response in most cases. However, the high treatment cost does not allow it to be used in all clinics [4, 5, 15].

Splenectomy is a second line of treatment when there is toxicity suspected. This treatment is considered by some authors as third-line treatment because it may induce premature labour. We consider that the spleen is the major area of platelet destruction in autoimmune thrombocytopenia. Thus, splenectomy leads to a high rate of durable complete remissions [1, 13, 16].

According to the ASH, the indication for splenectomy is a platelet count less than 10,000/mcL in the presence of haemorrhage in the second trimester of pregnancy, but there are few reports during the first trimester. During second trimester of pregnancy, the risks of anaesthesia are minimal to the foetus and uterine size does not complicate the procedure [17–19]. Laparoscopic splenectomy is currently recommended because it reduces the stay in bed, avoids other associated complications (i.e., thrombotic manifestations), and decreases the dose, frequency, and duration of analgesic treatment [13].

Alternative therapy such as azathioprine (FDA category D) has been used as an immunosuppressive agent during pregnancy without toxicity. Another option is rituximab (FDA category C), but its use for treating ITP during pregnancy has not been evaluated. However, it has been used for treatment of non-Hodgkin lymphoma during pregnancy [4, 5, 15].

ITP during pregnancy may be a clinical challenge when evidence is limited. Thus, clinicians should consider the risks and benefits of any proposed treatment plans and should obtain a multidisciplinary consensus.
